# Supplementary material for: Analyzing mixing systems using a new generation of Bayesian tracer mixing models
Source: PeerJ. 2018 Jun 21;6:e5096. doi: 10.7717/peerj.5096 (PMC6015753; doi:10.7717/peerj.5096)
Supplement: Supplemental Information 1 [file peerj-06-5096-s001.pdf]

MixSIAR Model Description

Contents

Preamble . . . 1
Source data . . . 1
  Raw source data . . . 2
  Mean / SD / n source data . . . 3
  Source data by factor . . . 3
Fixed / random / continuous effects . . . 3
  Fixed effects . . . 4
  Random effects . . . 4
  Choosing between fixed and random effects . . . 4
  Continuous effects . . . 5
  Nested vs. non-nested factors . . . 5
Mixture mean . . . 5
Mixture variance . . . 6
  Process x Residual error (with covariance) . . . 6
  Process x Residual error (no covariance) . . . 7
  Residual error only . . . 7
  Process error only . . . 7
Mixture likelihood . . . 7
References . . . 8

Preamble

MixSIAR incorporates many advances to mixing models since previous software packages (IsoSource, MixSIR, SIAR, IsotopeR) were written. While these advances were published elsewhere (Table 1), the novelty of MixSIAR is the integration of these into one framework. MixSIAR accomplishes this via the `write_JAGS_model` function, which constructs a JAGS model file (`MixSIAR_model.txt`) given the user's data structure and desired model options. This is not trivial because the model components interact. For example, the choice to include "Individual" as a random effect impacts the choice of error structure on the mixture. The type of source data (raw vs. mean/SD/n) determines whether you can fit the source data with covariance, and if this propagates through to the mixture covariance. As such, we describe the MixSIAR equations in a modular format. For the same reason, we also recommend that users save the JAGS model file and include it as a supplement to any publication, as this file specifies the equations used (there is no one "MixSIAR model").

Throughout, we use bold font to indicate multivariate variables (vectors, matrices), capital letters to indicate the total number of tracers (datapoints, sources, etc.), and lower case letters as indices. For instance, $\boldsymbol{p}$ is a vector of proportions of length K (total number of sources), and p_k indicates the proportion of the kth source.

Source data

Unlike MixSIR and SIAR, MixSIAR fits the source data hierarchically (also referred to as "fully Bayesian"). In other words, the model admits that the source means/SDs come from a sample and are not the truth. Therefore, the souce means/SDs used in the mixture likelihood, μ^s_{jk}, are allowed to deviate from the source sample means/SDs (and the amount of deviation depends on the source sample sizes; more deviation allowed for lower sample sizes). Note that in systems with lots of mixture data and few source data, the likelihood may be maximized by fitting the source means far from their sample means.

In order of preference (and model complexity), MixSIAR gives users three options to fit source data:

1. If a user has raw source data, MixSIAR includes *covariance* between tracers (preferred because it is the most complete "fully Bayesian" model).
2. If a user does not have raw source data and only provides summary statistics (mean, SD, and sample size), MixSIAR must assume tracers are independent (*no covariance*). For large datasets (500+ mixture data points), switching to mean/SD/n can reduce model runtime. This model is also "fully Bayesian" because it also estimates the 'true' source means and variances used in the mixture likelihood.
3. Users can effectively *turn off source fitting* by using the mean/SD/n option and changing the source sample size to an arbitrarily large number (i.e. set $n = 10000$). This will fix the source means at their sample means. In poorly resolved mixing systems (many sources, large SD, low n, few tracers), this can help the model converge. The resulting model is not "fully Bayesian", matching previous mixing model software (MixSIR, SIAR).

Raw source data

The data for tracer j, source k, Y^s_{jk}, are fit hierarchically as in Hopkins and Ferguson (2012) and Parnell et al. (2013):

$$\boldsymbol{Y^s_{jk}} \sim Normal\left(\mu^s_{jk}, \boldsymbol{\Sigma^s_k}\right),$$

The fitted source means, μ^s_{jk}, are used to construct the mixture likelihood. The priors on the source means are:

$$\mu^s_{jk} \sim Normal(0, 1000)$$

The source covariance matrices, Σ^s_k, are constructed as:

$$\boldsymbol{\Sigma^s_k} = diag\left(\omega^s_{jk}\right) \times \boldsymbol{\rho} \times diag\left(\omega^s_{jk}\right)$$

where the priors for the source precisions, $\frac{1}{{\omega^s_{jk}}^2}$, are:

$$\frac{1}{{\omega^s_{jk}}^2} \sim Gamma(.001, .001),$$

and the priors on the correlations between tracer i and tracer j are:

$$\rho_{ij} = \rho_{ji} \sim Uniform(-1, 1)$$

$$\rho_{ii} = 1$$

Option #1 should not be used for compositional tracer (e.g. fatty acid profile, FA) data, because the source data will not conform to the normality assumption. Therefore, we advise users with FA data to use option #2 described below. This is ok because the observed mixture tracer values are a (weighted) sum of random variables, which should be normally distributed according to the Central Limit Theorem (CLT). While FA data are not independent since each tracer must sum to 1, they are likely in the "sufficiently weakly correlated" class such that the CLT still holds. Article S2 demonstrates this via simulations.

Alternatively, analysts can use other software packages specifically designed to accommodate fatty acid data (QFASA, Iverson et al. 2004; fastinR, Neubauer & Jensen 2015).

Note: The diffuse Normal and Inv-Gamma priors on the source means, μ^s_{jk}, and variances, ${\omega^s_{jk}}^2$, work well for stable isotope and fatty acid tracers, because their order of magnitude is 10^{-1}-10^1. For other tracer types that are of larger orders of magnitude (e.g. element concentrations used in sediment mixing can be 10^3-10^5, Nosrati et al. 2014), these priors would not work. Instead of using the data to set the prior (i.e. by setting the prior mean equal to the sample mean), we scale the data so the same prior can be used regardless of data scale.

MixSIAR normalizes (subtract mean, divide by SD) the mixture and source tracer data before running the model. Normalizing the tracer data does not affect the proportion estimates, p_k, but does affect users seeking to plot the posterior predictive distribution for their data. For each tracer, we calculate the pooled mean and SD of the mix and source data, then subtract these pooled means and SDs from the mix and source data, and divide by the pooled SD. See lines 226-269 of `run_model.R`.

Mean / SD / n source data

In the event that a user does not have raw source data and only provides summary statistics (mean, variance, and sample size), we cannot fit the above model with covariance. Instead, we fit the source parameters μ^s_{jk} and Σ^s_k as in Ward et al. (2010):

$$\mu^s_{jk} \sim Normal\left(m_{jk}, n_k/s^2_{jk}\right),$$
$$tmp.X_{jk} \sim Chi\text{-}squared(n_k),$$
$$\frac{1}{{\omega^s_{jk}}^2} = \frac{tmp.X_{jk}}{s^2_{jk}\,(n_k - 1)},$$
$$\boldsymbol{\Sigma^s_k} = diag\left({\omega^s_{jk}}^2\right)$$

where:
m_{jk} = tracer j sample mean for source k (data),
s^2_{jk} = tracer j sample variance for source k (data),
n_k = source k sample size (data),
μ^s_{jk} = tracer j mean for source k (parameter),
${\omega^s_{jk}}^2$ = tracer j variance for source k (parameter),
$\boldsymbol{\Sigma^s_k}$ = source k covariance matrix (calculated from ω^s_{jk} terms).

Then, μ^s_{jk} and $\boldsymbol{\Sigma^s_k}$ are used in the mixture likelihood as for raw source data.

Source data by factor

If a user includes a factor as a fixed or random effect on the mixture data, MixSIAR allows the user to also include this factor on the source data. In this case, the source data for each factor level are fit independently and used in the mixture likelihood for mix datapoints in the same factor level. For example, in the wolves dataset (Semmens et al. 2009), the source data from Region 1 is used to fit the mixture data from Region 1, etc.

Fixed / random / continuous effects

In all cases, the overall ("global") source proportions, p_k, are drawn from:

$$\boldsymbol{p} \sim Dirichlet(\boldsymbol{\alpha})$$

where $\boldsymbol{p}$ and $\boldsymbol{\alpha}$ are vectors of length K, the number of sources. By default, the "uninformative" prior, $\alpha_k = 1$ for all k, is used. Users can specify their own informative prior using the `run_model` function. We then transform the global source proportions into ILR-space parameters, $\boldsymbol{\beta_0}$ (now a vector of length $k-1$), following Egozcue (2003):

$$\beta_{0k} = \sqrt{\frac{k}{k+1}}log\left(\frac{\sqrt[k]{\prod_{i=1}^{k} p_k}}{p_{k+1}}\right)$$

MixSIAR fits both fixed and random effects as offsets from the overall intercepts, $\boldsymbol{\beta_0}$, in ILR-space.

Fixed effects

The offset for the first level of a factor is set to 0 to avoid identifiability issues (i.e. first level, $\beta_{1_k}(1)$, becomes the intercept, $\boldsymbol{\beta_0}$):

$$\beta_{1_k}(1) = 0$$

Then for the remaining $L-1$ factor levels, the offset for the kth source for level l receives the prior:

$$\beta_{1_k}(l) \sim Normal(0, 1)$$

To get the proportion vector for the ith mixture, $\boldsymbol{p_i}$, we add the offsets for the factor level corresponding to mixture i, $\boldsymbol{\beta_1}(l_i)$, to the intercept, and then back-transform into p-space using the inverse ILR:

$$\boldsymbol{p_i} = ILR^{-1}\left[\boldsymbol{\beta_0} + \boldsymbol{\beta_1}(l_i)\right]$$

A second fixed effect can be added in the same way:

$$\beta_{2_k}(l) \sim Normal(0, 1)$$

$$\beta_{2_k}(1) = 0$$

$$\boldsymbol{p_i} = ILR^{-1}\left[\boldsymbol{\beta_0} + \boldsymbol{\beta_1}(l_i) + \boldsymbol{\beta_2}(l_i)\right]$$

With two fixed effects the intercept corresponds to the first level of factor 1 and the first level of factor 2.

Random effects

Random effects are added in much the same way, as offsets from the overall proportions with mean = 0:

$$\beta_{1_k}(l) \sim Normal(0, \gamma_1^2)$$

$$\gamma_1 \sim Uniform(0, 20)$$

Choosing between fixed and random effects

MixSIAR fits both fixed and random effects as offsets from the overall intercepts, $\boldsymbol{\beta_0}$, in ILR-space. We recognize that the terms "fixed" and "random" effects are unclear (Gelman 2005), and in Gelman's *constant* versus *varying* terminology, both fixed and random effects in MixSIAR are *varying* (different for each factor level l). In MixSIAR, for a categorical factor with L levels in a model with K sources:

	**Fixed effect**	**Random effect**
(Effective) parameters added	$(L-1)(K-1)$	$< L(K-1)+1$<br>$> K$
Relationship between levels	Independent	Hierarchical
Equations	$\beta_{1_k}(1) = 0$<br>$\beta_{1_k}(l) \sim Normal(0,1)$	$\beta_{1_k}(l) \sim Normal(0, \gamma_1^2)$<br>$\gamma_1 \sim Uniform(0, 20)$

There are two practical distinctions between these models:

1. *Number of (effective) parameters added*: the fixed effects version generally has more, but it depends on the dataset. The number of parameters will be greater in the random effects version, but the *effective* number of parameters is often lower because they share information (Gelman et al. 2004). The random effects model adds one parameter for each factor, γ_1, but the effective number of β_{1_k} is between L (one

for each level) and 1 (mean for all levels). Using the wolves example (Semmens et al. 2009), estimating Pack offsets as fixed effects results in $(8-1)(3-1) = 14$ additional parameters, while the random effects model adds somewhere between 3 and $8(3-1)+1 = 17$. Thus, if the factor has many levels, it may be better to use the random effects model. If the factor has few levels (< 5), however, it can be difficult to estimate the random effect variance term (γ_1^2). If the factor has only 2 levels (e.g. Sex), γ_1^2 cannot be estimated and the factor should be treated as a fixed effect.

2. *Independence*: the random effects model draws offsets from a shared distribution, which makes sense if the factor levels are related. Since hierarchical structure is common in biological systems, random effects often make sense. If the factor levels are truly independent, then treating the factor as a fixed effect may be best.

Continuous effects

We add continuous effects as linear terms in ILR-space. Before model fitting, we standardize the covariate (subtract mean and divide by standard deviation, see `load_mix_data`).

$$\beta_{1_k} \sim Normal(0, 1000)$$

To get the source proportions for mixture i, we multiply the linear terms, $\boldsymbol{\beta_1}$, by the value of the covariate for mixture i, x_i:

$$\boldsymbol{p_i} = ILR^{-1}\left[\boldsymbol{\beta_0} + \boldsymbol{\beta_1} x_i\right]$$

Nested vs. non-nested factors

When two categorial factors are included, the user must tell MixSIAR whether the factors are independent or nested within each other. This affects the calculation of proportions at the factor level. To use the wolves example, each Pack (β_2) is nested within a Region (β_1). Therefore, to calculate the proportions for pack l:

$$\boldsymbol{p_l} = ILR^{-1}\left[\boldsymbol{\beta_0} + \boldsymbol{\beta_1}(l) + \boldsymbol{\beta_2}(l)\right]$$

where $\boldsymbol{\beta_1}(l)$ is the Region offset where Pack l is found, and $\boldsymbol{\beta_2}(l)$ is the Pack offset from the Region mean.

If we change this example such that factor 2 is *not* nested within factor 1 (e.g. Species that are found in each Region, Region and Species treated independently), then MixSIAR calculates the proportions for the lth level of factor 2 without adding the offset for factor 1:

$$\boldsymbol{p_l} = ILR^{-1}\left[\boldsymbol{\beta_0} + \boldsymbol{\beta_2}(l)\right]$$

Mixture mean

MixSIAR assumes mass balance and calculates the mixture mean for each datapoint, μ_{ij}^m, as a convex combination of the source proportions, $\boldsymbol{p_i}$, times the *fitted* source means, μ_{jk}^s, adjusted by the mean TDF, λ_{jk}, and concentration of tracer j in source k, q_{jk}:

$$\mu_{ij}^m = \frac{\sum_k p_{ik} q_{jk} (\mu_{jk}^s + \lambda_{jk})}{\sum_k p_{ik} q_{jk}}$$

This is the same as previous mixing models (IsoSource, MixSIR, SIAR, IsotopeR, etc.)

Mixture variance

There are three user options for the mixture variance. For motivation, description, and simulation test results, see Stock and Semmens (2016). In brief:

1. Process x Residual error (default)
    a. with covariance (if raw source data)
    b. without covariance (if source means/SD/n)
2. Residual error

Situations where the true variation in source tracer values is not reflected in the mixture data (e.g. integrated sampling), or it does not make sense to think of the consumers (mixtures) sampling individual prey (source) items (e.g. oysters filter feeding, sediment fingerprinting). For large datasets (500+ mixture data points), switching to the residual error model can reduce model runtime.

3. Process error

Required when there is only one mixture datapoint (not possible to fit mixture variance term), or one mixture datapoint per fixed/random effect level. Including “ID” or “Individual” as a fixed/random effect is an important example where this applies.

Process x Residual error (with covariance)

The source proportions for individual i, $\boldsymbol{p_i}$, fitted source covariance, $\boldsymbol{\Sigma_k^s}$, and TDF covariance, $\boldsymbol{\Sigma_k^c}$, are used to construct the mixture covariance for individual i, $\boldsymbol{\Sigma_i}$:

$$\boldsymbol{\Sigma_i} = \boldsymbol{\Sigma^{res}} \odot \sum_k p_{ik}^2 \left(\boldsymbol{\Sigma_k^s} + \boldsymbol{\Sigma_k^c}\right)$$

The TDF variance of tracer j for source k, τ_{jk}^2, is input by the user:

$$\boldsymbol{\Sigma_k^c} = diag\left(\tau_{jk}^2\right)$$

There is one multiplicative error term, ϵ_j, fit for every tracer:

$$\epsilon_j \sim Uniform(0, 20)$$

$$\boldsymbol{\Sigma^{res}} = \begin{cases} \epsilon_i & \text{for } i = j, \\ \sqrt{\epsilon_i \epsilon_j} & \text{for } i \neq j \end{cases}$$

For example, if there are j=2 tracers:

$$\boldsymbol{\Sigma_i} = \boldsymbol{\Sigma^{res}} \odot \sum_k p_{ik}^2 \left(\boldsymbol{\Sigma_k^s} + \boldsymbol{\Sigma_k^c}\right) = \begin{bmatrix} \epsilon_1 & \sqrt{\epsilon_1 \epsilon_2} \\ \sqrt{\epsilon_1 \epsilon_2} & \epsilon_2 \end{bmatrix} \odot \begin{bmatrix} \sum_k p_{ik}^2 \left(\omega_{1k}^2 + \tau_{1k}^2\right) & \sum_k p_{ik}^2 \rho \omega_{1k} \omega_{2k} \\ \sum_k p_{ik}^2 \rho \omega_{1k} \omega_{2k} & \sum_k p_{ik}^2 \left(\omega_{2k}^2 + \tau_{2k}^2\right) \end{bmatrix}$$

$$= \begin{bmatrix} \epsilon_1 \sum_k p_{ik}^2 \left(\omega_{1k}^2 + \tau_{1k}^2\right) & \sqrt{\epsilon_1 \epsilon_2} \sum_k p_{ik}^2 \rho \omega_{1k} \omega_{2k} \\ \sqrt{\epsilon_1 \epsilon_2} \sum_k p_{ik}^2 \rho \omega_{1k} \omega_{2k} & \epsilon_2 \sum_k p_{ik}^2 \left(\omega_{2k}^2 + \tau_{2k}^2\right) \end{bmatrix}$$

Note: $\odot$ denotes element-wise multiplication, and ρ and ω_{jk}^2 are the correlation and variances of the source covariance matrix, $\boldsymbol{\Sigma_k^s}$.

Process x Residual error (no covariance)

When there is no information on the covariance of the sources (i.e. user inputs mean/SD/n data), the off-diagonal entries of $\boldsymbol{\Sigma_k^s}$ are 0. Then the mixture variance is:

$$\boldsymbol{\Sigma_i} = \boldsymbol{\Sigma^{res}} \odot \sum_k p_{ik}^2 \left(\boldsymbol{\Sigma_k^s} + \boldsymbol{\Sigma_k^c}\right)$$

$$\boldsymbol{\Sigma_i} = diag\left(\epsilon_j \sum_k p_{ik}^2 \left(\omega_{jk}^2 + \tau_{jk}^2\right)\right)$$

$$\epsilon_j \sim Uniform(0, 20)$$

E.g. for j=2,

$$\boldsymbol{\Sigma_i} = \begin{bmatrix} \epsilon_1 \sum_k p_{ik}^2 \left(\omega_{1k}^2 + \tau_{1k}^2\right) & 0 \\ 0 & \epsilon_2 \sum_k p_{ik}^2 \left(\omega_{2k}^2 + \tau_{2k}^2\right) \end{bmatrix}$$

Residual error only

Sometimes the true variation in source tracer values is not reflected in the mixture data (e.g. integrated sampling), or it does not make sense to think of the consumers (mixtures) sampling individual prey (source) items (e.g. oysters filter feeding, sediment fingerprinting). In these cases, the source and mixture data do not follow the standard assumptions about the mixing process (see discussion of "process" vs. "residual" error in Stock and Semmens (2016)). Since there is no information about the source variance, MixSIAR directly fits the mixture variance, $\boldsymbol{\Sigma}$, as a residual error term with a Wishart prior:

$$\boldsymbol{\Sigma} \sim InvWish\left(\boldsymbol{I}, j+1\right)$$

where $\boldsymbol{I}$ is the identity matrix, and j is the number of tracers.

Process error only

When there is only one mixture datapoint (or one mixture datapoint per fixed effect level), it is not possible to fit a mixture variance term. In order to define the likelihood of the one mixture datapoint, MixSIAR assumes that the mixture variance is defined by the proportions and the source variances (i.e. the distribution of mixture tracer data is the mathematical result of adding k independent normal random variables, the sources):

$$\sigma_j^2 = \sum_k p_k^2 \left(\omega_{jk}^2 + \tau_{jk}^2\right)$$

$$\boldsymbol{\Sigma} = diag\left(\sigma_j^2\right)$$

Mixture likelihood

Once the mixture mean and covariances are calculated as above, the likelihood for the data from consumer i and tracer j, Y_{ij} is:

$$Y_{ij} \sim Normal\left(\mu_{ij}^m, \boldsymbol{\Sigma_i}\right)$$

Table 1: Previously described mixing model advances that are implemented in MixSIAR.

**Process**	**Description**	**Reference**
Process error	Sampling error of true source values (*consumer*)	Moore and Semmens (2008)
Fractionation error	Consumers *differentially process* source tissue	Moore and Semmens (2008)
Hierarchical source fitting	Sampling error of true source values (*scientist*)	Ward et al. (2010)
Individual (random) effect	Individual mixtures *randomly* deviate from mean	Semmens et al. (2009)
Random/fixed effects	Individual mixtures deviate from mean *in a predictable way, by a categorical covariate*	Semmens et al. (2009)
Continuous effects	Individual mixtures deviate from mean *in a predictable way, by a continuous covariate*	Francis et al. (2011)
Concentration dependence	*Sources contribute variably* to each tracer value in a consumer's tissue	Phillips and Koch (2002)
Residual error	*Unexplained* variability of mixture data	Parnell et al. (2010)
Covariance	Tracers *covary* in source/mixture data	Hopkins and Ferguson (2012)
Process x Residual error	Mixture variance term motivated by mixing process	Stock and Semmens (2016)

References

Egozcue, JJ, V Pawlowsky-Glahn, G Mateu-Figueras, and C Barcelo-Vidal. 2003. "Isometric logratio transformations for compositional data analysis." *Mathematical Geology* 35 (3): 279–300. doi:10.1023/A:1023818214614.

Francis, TB, DE Schindler, GW Holtgrieve, ER Larson, MD Scheuerell, BX Semmens, and EJ Ward. 2011. "Habitat structure determines resource use by zooplankton in temperate lakes." *Ecology Letters* 14 (4): 364–72. doi:10.1111/j.1461-0248.2011.01597.x.

Gelman, A. 2005. "Analysis of variance–Why it is more important than ever." *The Annals of Statistics* 33 (1): 1–53. doi:10.1214/009053604000001048.

Gelman, A, JB Carlin, HS Stern, and DB Rubin. 2004. *Bayesian Data Analysis*. 2nd ed. Boca Raton, FL: CRC Press.

Hopkins, JB, and JM Ferguson. 2012. "Estimating the diets of animals using stable isotopes and a comprehensive Bayesian mixing model." *PLoS ONE* 7 (1): e28478. doi:10.1371/journal.pone.0028478.

Moore, JW, and BX Semmens. 2008. "Incorporating uncertainty and prior information into stable isotope mixing models." *Ecology Letters* 11 (5): 470–80. doi:10.1111/j.1461-0248.2008.01163.x.

Nosrati, K, G Govers, BX Semmens, and EJ Ward. 2014. "A mixing model to incorporate uncertainty in sediment fingerprinting." *Geoderma* 217: 173–80. doi:10.1016/j.geoderma.2013.12.002.

Parnell, AC, R Inger, S Bearhop, and AL Jackson. 2010. "Source partitioning using stable isotopes: Coping with too much variation." *PLoS ONE* 5 (3): 1–5. doi:10.1371/journal.pone.0009672.

Phillips, DL, and PL Koch. 2002. "Incorporating concentration dependence in stable isotope mixing models." *Oecologia* 130: 114–25. doi:10.1007/S004420100786.

Semmens, BX, EJ Ward, JW Moore, and CT Darimont. 2009. "Quantifying inter-and intra-population niche variability using hierarchical bayesian stable isotope mixing models." *PLoS ONE* 4 (7): 1–9. doi:10.1371/journal.pone.0006187.

Stock, BC, and BX Semmens. 2016. "Unifying error strucctures in commonly used biotracer mixing models." *Ecology* 97 (3): 576–82. doi:10.1002/ecy.1517.

Ward, EJ, BX Semmens, and DE Schindler. 2010. "Including source uncertainty and prior information

in the analysis of stable isotope mixing models." *Environmental Science & Technology* 44 (12): 4645–50. doi:10.1021/es100053v.
