# Supplementary material for: Analyzing mixing systems using a new generation of Bayesian tracer mixing models
Source: PeerJ. 2018 Jun 21;6:e5096. doi: 10.7717/peerj.5096 (PMC6015753; doi:10.7717/peerj.5096)
Supplement: Supplemental Information 2 [file peerj-06-5096-s002.pdf]

Is transforming compositional tracer (e.g. fatty acid) data necessary?

MixSIAR assumes that the mixture data is a weighted sum of the source means:

$$Y_{ij} \sim Normal\left(\sum_k p_{ik}\mu^s_{jk}, \Sigma\right)$$

Because the mixture data for each tracer j, Y_{ij}, is a sum of random variables, they should be normally distributed according to the Central Limit Theorem (CLT). While compositional tracer (e.g. fatty acid) data are not independent since they sum to 1, they are probably in the "sufficiently weakly correlated" class such that the CLT still holds. Here we test this assumption via simulation.

Construct vector p representing contribution of K=5 sources

```
library(MCMCpack)
library(compositions)
library(tidyverse)
K = 5
alpha=rep(1/K, K)
p = MCMCpack::rdirichlet(1, alpha)
# don't allow any true p < 0.05
# mixing models assume all sources contribute to mixture
while(any(p < .05)){ p = MCMCpack::rdirichlet(1, alpha)}
```

Set the number of tracers, J, to 10 (FA datasets often have 10-20 tracers). Use dirichlet to force tracer data to be compositional (as for FA, all between 0-1 and sum to 1 – not independent).

Create matrix of means for the K=5 sources and J=10 tracers

```
J = 10 # number of tracers
alph2 <- rep(1/J, J)
m = MCMCpack::rdirichlet(K, alph2) # tracer means for each source sum to 1
```

Simulate p for 10,000 mixtures with Unif(-0.5,0.5) residual error added in ILR-space

```
options(warn = -1) # compositions package displays annoying warnings
jitter_p <- function(p, N=10000){
  k <- length(p)
  # devs <- matrix(rnorm((k-1)*N, 0, 0.2), N, k-1) # normal error in ILR-space
  devs <- matrix(runif((k-1)*N, -0.5, 0.5), N, k-1) # uniform error in ILR-space
  jittered <- apply(devs, 1, function(x) compositions::ilrInv(compositions::ilr(p) + x))
  return(t(jittered))
}
jittered.p <- jitter_p(p)
```

```r
# True mix proportions
p
```

```
##              [,1]      [,2]      [,3]      [,4]      [,5]
## [1,] 0.2704288 0.3689649 0.1058024 0.1522066 0.1025974
```

```r
# Individual mix proportions
head(jittered.p)
```

```
##              1         2          3         4          5
## [1,] 0.2882055 0.2362215 0.12761065 0.2213194 0.12664299
## [2,] 0.2258739 0.5257183 0.06378454 0.1210788 0.06354443
## [3,] 0.2063639 0.4684197 0.07392977 0.1943296 0.05695700
## [4,] 0.1582320 0.3656847 0.10801631 0.2020463 0.16602068
## [5,] 0.3970917 0.3755009 0.07157534 0.1005436 0.05528851
## [6,] 0.2914644 0.2592881 0.14088365 0.2316562 0.07670764
```

Calculate mixture data as weighted averages

```r
Y = jittered.p %*% m
```

Histograms of mix data by tracer

```r
longY <- Y %>% as.data.frame %>% gather(key="tracer", value="FAval")
longY$tracer <- factor(longY$tracer, labels=paste0("Tracer ", 1:J))
ggplot(longY, aes(x=FAval, group=tracer)) +
  geom_histogram() +
  facet_wrap(~tracer, scales = "free") +
  theme_classic()
```

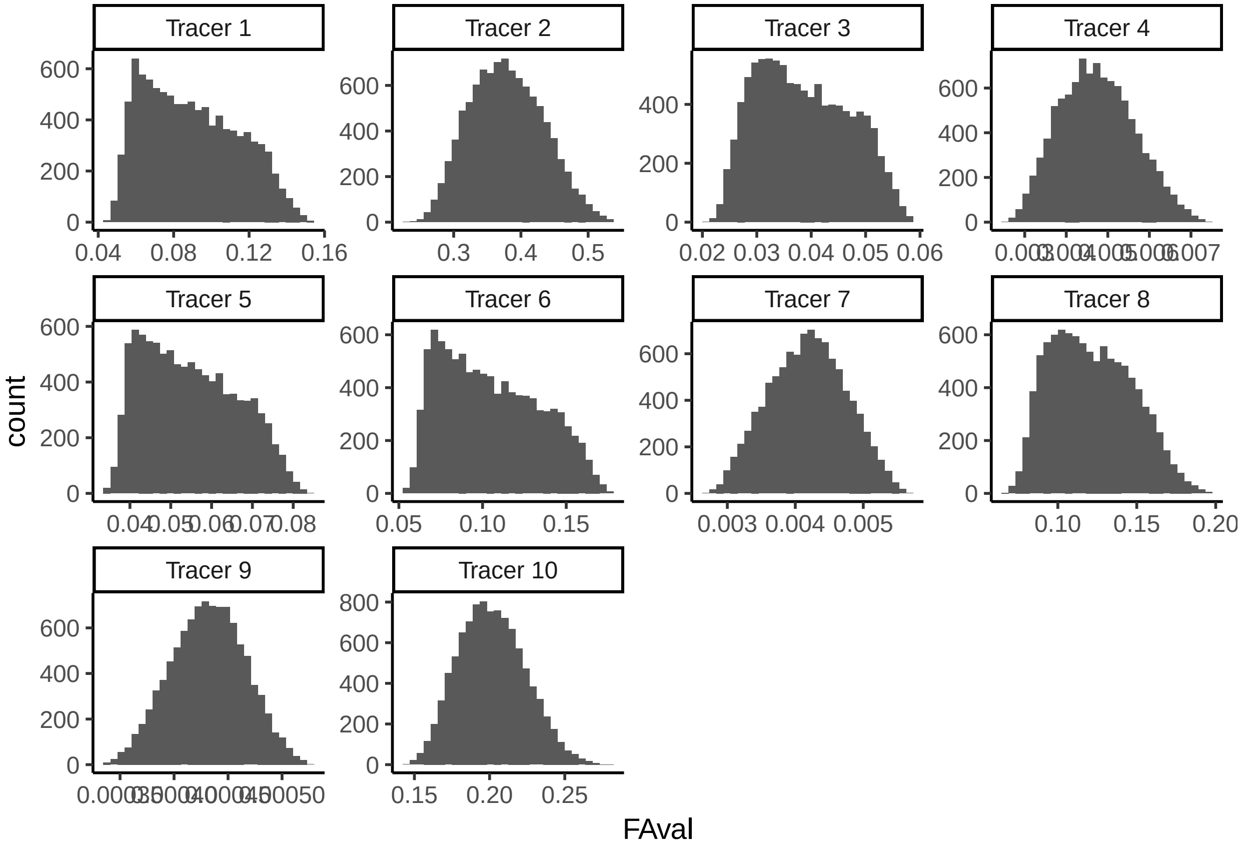
Tracer 1
Tracer 2
Tracer 3
Tracer 4
Tracer 5
Tracer 6
Tracer 7
Tracer 8
Tracer 9
Tracer 10
count
FAval
